# Injuries of the upper cervical spine: A series of 28 cases

**DOI:** 10.4103/0019-5413.36992

**Published:** 2007

**Authors:** Saumyajit Basu, Sandip Chatterjee, Manoj Kumar Bhattacharya, Kaushik Seal

**Affiliations:** Park Clinic and Kothari Medical Center, Kolkata, India

**Keywords:** Upper cervical spine, occipital condyle, hangman's fracture, atlanto - axial dislocation

## Abstract

**Background:**

There are very few published reports of upper cervical spine injuries from our country and there is a heavy bias towards operative treatment of these injuries. We present below our experience of upper cervical injuries over the last four years.

**Materials and Methods:**

Twenty eight patients (20 males, 8 females) with upper cervical spine injury (including Occiput, Atlas and Axis) were treated and were followed-up for an average of 11.2 months. The data was analyzed retrospectively with regards to the location and type of injury, the treatment offered (conservative or operative) as well as the final clinical and radiological outcome.

**Results:**

The clinico-radiological outcome of treatment of these injuries is mostly very good with few complications. Other than a single case of mortality due to associated head injury there were no major complications.

**Conclusion:**

Management of these patients needs a proper evaluation to arrive at the type of injury and prompt conservative or operative treatment. Treatment is usually safe and effective with good clinical and radiological outcome.

Upper cervical spine injuries account for about 24% of acute fractures and dislocations of the cervical spine.^1^ There is a paucity of published reports on upper cervical spine injuries from our country. We report an analysis of a series of 28 cases of traumatic upper cervical injuries highlighting the diagnosis, clinical and radiological evaluation, treatment and outcome.

## MATERIALS AND METHODS

The present retrospective study consists of 28 patients (20 males, 8 females) of upper cervical spine injuries, whose complete data including the case notes, X-rays/CT scans/MRI and followup notes was analyzed. The following was the distribution of the injuries [Table 1].

**Table 1:** Distribution of upper cervical spine injury cases

Atlanto occipital injuries	Occipital condyle fractures	1
Atlanto occipital dislocations		0
Atlas (C1) fractures	Type I/II/III	5
Atlanto axial dislocations	Transverse ligament ruptures	0
	C1/C2 rotary subluxation	4
Axis (C2) fractures	Odontoid fractures	13
	Hangman fractures	5

Twenty three out of 28 patients presented early (within the first 3 weeks) and and five presented late (after 3 weeks) out of which two were untreated C1/C2 rotary subluxation, two were neglected/untreated Type II odontoid fractures and one was a case of C2/3 dislocation [Table 2]. The patients presented with severe neck pain and stiffness with restricted movements in all direction. Radicular pain and brachialgia was absent in all. Neural deficits in the form of spastic quadriparesis was found in three patients who presented late. Hemiparesis was observed in one Type II odontoid fracture with severe head injury. No patient had cranial nerve palsies though they were examined in all. Five patient had evidence of head injury. Three patients had associated minor scalp injuries and one patient had associated facial soft tissue injury.

**Table 2:** showing details of the patients of upper cervical spine injury

Name	Age (yr)	Sex	Location of injury	Type	Treatment	Complications	FU (mths)
AC	56	Female	Atlas # +Type II Odontoid #	Combined	Occipito-cervical fusion (Contoured Rod+sublaminar wires)	Nil	13
DE	44	Male	Atlas fracture	Type II	Halo-vest	Pin loosening	9
GT	34	Female	Atlas fracture	Type III	Halo-vest	Nil	48
VC	22	Male	Atlas fracture	Type III	Halo-vest	Pin loosening	14
FR	56	Male	Atlas fracture	Type I	SOMI brace	Nil	8
CB	14	Female	C1-C2 rotary subluxation	Type III	Posterior C1/C2 fusion with Transarticular screws	Nil	17
BC	6	Male	C1-C2 rotary subluxation	Type III	Posterior C1/C2 fusion with Transarticular Screws	Nil	7
CC	9	Female	C1-C2 rotary Subluxation	Type I	Halo-vest	Nil	10
AB	12	Male	C1-C2 rotary subluxation	Type I	SOMI brace	Nil	9
SB	62	Male	C2-C3 dislocation	? Variant of Type III	Ant, C2/3 discectomy fusion (locking plate-screw fixation)	Prolonged swallowing difficulty	8
KK	67	Male	Hangman fracture	Type IIa	Ant, C2/3 discectomy fusion (locking plate-screw fixation)	Nil	11
UF	34	Male	Hangman fracture	Type III	Ant, C2/3 discectomy fusion (locking plate-screw fixation)	Nil	7
RR	45	Female	Hangman fracture	Type III	Ant, C2/3 discectomy fusion (locking plate-screw fixation)	Nil	9
PL	34	Male	Hangman fracture	Type Iia	SOMI brace	Nil	9
SD	22	Male	Occipital condyle	Type I	SOMI brace	Nil	16
IB	57	Male	Odontoid fracture	Type II displaced	Post decomp + OC Fusion (Contoured Rod + sublam wires)	Nil	12
JB	33	Male	Odontoid fracture	Type II displaced	Anterior Odontoid Screew Fixation	Nil	8
KB	45	Female	Odontoid fracture	Type II displaced	Anterior Odontoid Screew Fixation	Nil	12
NB	24	Male	Odontoid fracture	Type II undisplaced	Halo-vest	Pin loosening	8
DP	43	Male	Odontoid fracture	Type III	Halo-vest	Nil	7
MB	22	Male	Odontoid fracture	Type II displaced	Post C1/C2 fusion with Sublaminar Cable (Brooks/Jenkins)	Nil	9
NB	16	Male	Odontoid fracture	Type II displaced	Posterior C1/C2 fusion with Transarticular Screws	Nil	10
PK	32	Female	Odontoid fracture	Type II displaced	Posterior C1/C2 fusion with Transarticular Screws	Nil	7
IG	34	Male	Odontoid fracture	Type II displaced	Posterior C1/C2 fusion with Transarticular Screws	Delayed brain stem omplications - death	9
TG	33	Female	Odontoid fracture	Type II displaced	Posterior C1/C2 fusion with Transarticular Screws	Nil	11
YB	41	Male	Odontoid fracture	Type II displaced	Postrior C1/C2 fusion with Wire (Gallie)	Nil	8
TB	60	Male	Odontoid fracture	Type III	SOMI brace	Nil	11
MK	26	Male	Odontoid fracture	Type II displaced	Transoral decomp, +Post.decomp +OC fusion (cont. rod+sl wires)	Nil	7

Radiological evaluation included cervical spine X-rays (open mouth AP and Lateral). Supervised flexion/extension lateral X-rays were done in all cases. However, they were avoided initially in those cases of associated neural deficit and in the patient with major head injury. CT-scans of the cervical spine with thin slices at C1, C2 region and good quality saggital, coronal and three-dimensional reconstructions were done in all patients. MRI of the cervical spine was also done to rule out neural compression and to look for evidence of cord edema or gliosis. We did CT scan of the brain in all patients with associated head injury.

The fresh cases were put on cervical immobilization as soon as they arrived in hospital with Philadelphia collar and were log rolled. Glasgow Coma Scale score was documented periodically with all the patients of head injury. Out of the five patients of head injury, four were minor head injuries without major findings on the CT scan. However one patient had a major brain stem injury and frontal contusion along with odontoid fracture.

### Atlanto occipital injuries

We had a single patient of occipital injury, which was a Type I (impacted) fracture and was treated with a SOMI brace for three months without further complications.

### Atlas (C1) fractures

**Figure 1:**
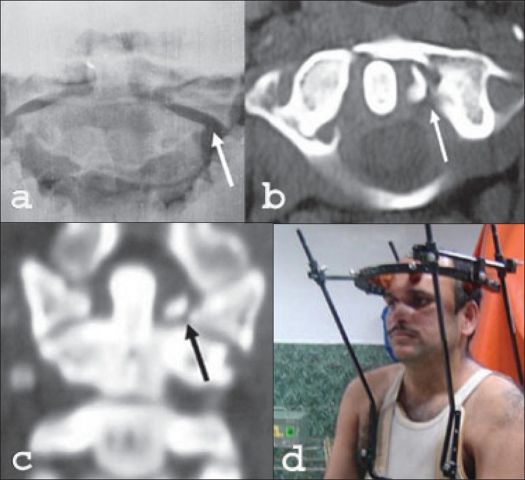
32 years old male with Jefferson fracture. Open mouth x-ray (a) shows lateral subluxation of C1-C2 joint (white arrow). Axial CT scan (b)on the left side (white arrow) and coronal reconstruction (c) (black arrow) shows the lateral subluxation. (d) The patient was treated with halo vest immobilization

Five patients had Atlas Fracture. Four presented with history of road traffic accidents while one, fell from a staircase. None of them had any neural deficit but had severe neck pain and stiffness. Four of them were isolated atlas fractures and one with a combined posterior arch of atlas and Type II Odontoid fracture. Of the four patients with isolated atlas fractures, one was Type I, one was Type II and two were Type III (Jefferson fracture).^3^,^4^ Both the Jefferson fractures and the patient with lateral mass fracture (Type II) were treated with Halo-vest immobilization for six weeks followed by SOMI brace for another six weeks [Figure 1]. The lone patient with isolated anterior arch fracture (Type I) was treated with SOMI brace immobilization for 12 weeks. All of them had an uneventful recovery. After removal of immobilization they took about two months to regain normal painless arc of motion and there were no significant complications other than pin-site infections in two of the halo-immobilized patients. Fresh pins were put through adjacent holes in these without further problems. At one year followup they all did well.

One patient had with a combined posterior arch of atlas fracture and Type II Odontoid fracture (grossly unstable though reducible on dynamic x-rays). This combination occurs quite often.^5^ This patient presented two months after the trauma with persistent radiological instability. The posterior occipitocervical fixation and fusion with contoured rod and sublaminar wires in occiput (C0) to the axis (C2) was performed. He went on to have good radiologically evident C0 to C2 posterior fusion and was completely painless at last followup at two years postoperatively. There was about 40% loss of cervical range of motion.

### Atlanto – axial dislocations

We did not have any patient with pure traumatic Atlanto - Axial Dislocations. No patient had pure transverse ligament intra-substance (Type I) rupture though we had one case of avulsion injury at the insertion of transverse ligament on the inner aspect of the lateral mass (Type II). This patient had a lateral mass fracture of the atlas and was treated conservatively with halo-vest immobilization (as described above) and it healed completely without residual problems.

Four patients had C1-C2 Rotary Subluxation out of which two presented early (within a week of injury) and were Type I injuries^6^ with anterior Atlanto - Dens Interval (ADI) on X-rays less than 3 mm. These patients were put on halo vest immobilization after initial skull traction for one week. One of them refused a Halo and so we had to put a SOMI. After 6 weeks, the halo was removed and a SOMI brace was used for another six weeks. Both of them made a good uneventful recovery.

**Figure 2:**
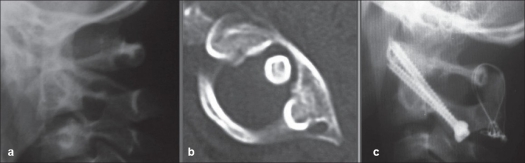
Lateral x-ray of upper cervical spine (a) and CT scan (b) shows type III C1-C2 rotatory subluxation 22 days after injury in a 14 year old girl. The atlanto-dens interval is about 6 mm. she was initially put on skull traction for reduction and then C1-C2 fusion was done with transarticular screws. Followup lateral x-rays (c) shows trasnarticular screw and C1-C2 fusion

The remaining two presented late (one after three weeks and the other after four weeks) and were Type III injures with ADI on flexion X-rays more than 5 mm. Both these patients were put on skull traction for initial two weeks followed by posterior C1-C2 fusion with fixation by transarticular screws [Figure 2]. In one reduction was partial and one screw could be put in (C1, C2 sublaminar wiring was added) though he became painless after fusion was completed. Torticollis was corrected in both of them, in the later with partial reduction, possibly due to realignment and compensatory adjustment of the distal joints.

### Axis (C2) fractures

We had 13 patients of odontoid fractures -11 of Type II, 2 of Type III.^7^ One undisplaced Type III fracture was treated with SOMI brace for six weeks followed by six weeks of hard collar usage. The remaining Type III and one undisplaced fracture of Type II was treated conservatively with halo vest immobilization for six weeks followed by SOMI brace for another six weeks. Out of the remaining 10 patients of displaced Type II fractures two patients had fresh, reducible, transverse and non-comminuted fractures and they were operated upon with anterior odontoid screw fixation (4 mm cannulated cancellous lag screws, one in number) and primary osteosynthesis in one and with C1-C2 posterior spinal fusion with wire in another. Six of the remaining eight underwent C1-C2 posterior fusion and two underwent occipitocervical fusion. All of those who underwent posterior C1-C2 fusions had displaced and/or irreducible Type II fractures, which were either severe by comminution (Type IIa Hadley^8^; n=4) or presented late (after four weeks; n=2). The instrumentation used in posterior fusion was C1-C2 transarticular screw (Magerl) in four and wire/cable in two (Gallie/Brooks).

One of the four patients who underwent C1-C2 transarticular screw fixation had a severely comminuted unstable Type II fracture of the odontoid and on MRI had gross cord edema extending to the brain stem along with head injury in the form of frontal contusion though there was no initial mid-line shift on the CT scan. He woke up fine after the surgery and was well for the first three days but then had a deteriorating level of consciousness. He ultimately required ventillatory support and died from septicaemia leading to end-stage multi-organ failure three weeks after the surgery.

In the remaining two patients, occipitocervical fusion was done. One was a two-year-old comminuted unstable Type II fracture who presented with myelopathy where we performed posterior decompression (which included excision of posterior arch of atlas because of the compression shown on the MRI) and contoured rod fixation with sublaminar wires in C0 and C2 along with posterior iliac crest bone grafting. The other was a similar case in which the compression was from the front and back. This patient underwent an anterior transoral odontoidectomy and posterior decompression with occipitocervical fixation (contoured rod and sublaminar wires in C0 and C2) and fusion in single stage. Both these patients recovered well. Neurological improvement was seen in both and went up from Frankle Grade C to Grade D.

We had four patients of Hangman's fractures; two were Type IIa with and two were Type III.^9^ One patient had isolated C2/3 dislocation, who presented 10 days after the trauma. Both patients of Type III and one patient of Type IIa had anterior C2/3 discectomy with locking plate-screw fixation and fusion (one patient of Type IIa was conserved with SOMI brace because he refused halo-vest) while the patient with C2/3 dislocation was initially treated with skull traction for five days leading to partial reduction followed by anterior discectomy, locking plate-screw fixation and fusion. This patient had a prolonged swallowing difficulty for three weeks after which it settled on its own. Upper GI endoscopy and barium swallow was negative in this patient.

## RESULTS

In our series we had 28 patients (20 males and 8 females) of upper cervical injuries with an average follow up of 11.2 months (range 6 to 48 months). Out of these we had one patient of occipital condylar fracture, five patients of C1 fractures, four patients of C1/C2 rotary subluxation, 18 patients of C2 fractures (13 patients of odontoid fractures, four patient of Hangman's fracture and one patient of C2/C3 dislocation).

Conservative treatment was offered to a total of 11 patients and all of them did well without any major complications till the last follow up. Six of them were treated with Halo Vest Immobilization followed by SOMI brace. The remaining five patients were treated with six weeks of SOMI usage followed by six weeks of hard cervical collar. Out of the six patients treated by Halo vest immobilization; there were three cases of pin loosening. All of them were treated with change of pin-site without any further problems. Pin-site care was taught to the parents/care-providers as was appropriate. There was one case of pressure sore developing from a SOMI brace, which gradually healed after the brace was taken off.

Operative treatment was offered to a total of 17 patients. There was a single case of mortality due to severe cord and brain stem edema, which occurred at the time of injury which ultimately required ventilatory support leading to multi-organ failure. There was one patient of prolonged swallowing difficulty in an anterior C2/C3 fusion, which ultimately resolved completely within two weeks after surgery. There were no other major complications in the rest 15 of them till the last followup. There was no iatrogenic neural injury or wound problems. There was no infection recorded in our operative cases.

All patients were followed-up regularly with clinical and radiological evaluation. There was no tenderness in any of these patients. Neck movements were well maintained excepting the patients who had occipitocervical fusion in which there was about 40% loss of movement, especially rotation. Neural deficit was present in only two cases of delayed presentations of Type II odontoid fractures. Both these had myelopathy at the time of presentation, as discussed above and both had improvement by one grade. Both became independent walkers. There was some persistent evidence of spasticity in both. The only other patient who had spasticity before surgery (he had C2/3 dislocation) recovered completely. X-rays were taken at three months and 12 months, including flexion/extension lateral views of the cervical spine. None showed perceptible abnormal movement. Repeat CT scans were suggested to all of them at one year followup but unfortunately none agreed, citing economic constraints. Overall clinical and radiological features are very satisfactory.

## DISCUSSION

Upper cervical spine injuries have been described to account for about a quarter of cervical spine injuries. While occipital condylar fractures are quite rare, they are treated conservatively in most instances. Anderson^2^ described three types of occipital condylar fractures type I - impaction; type II - basilar skull fracture; and type III - avulsion fracture. Types I and II are stable and are treated with rigid external cervical immobilization, preferably a halo vest.

Atlas fractures are commoner and are usually treated conservatively. Lateral mass fractures or Jefferson fractures may involve a rupture of the transverse ligament especially if the lateral translation of the C1/C2 joint is more than 7 mm.^10^ We had a single patient with ruptured transverse ligament [Figure 1] but he did well with halo vest immobilization. Though the reported incidence of combined fracture of C1 and C2 has been reported to be 53%,^5^ we had only one of the five injuries. This patient presented late with persistent nonunion of the dens fracture for which an instrumented C0-C2 fusion was performed.

Pure transverse ligament rupture, we believe is much more often associated with congenital (Down's syndrome, hypoplastic odontoid, os odontoideum, assimilation of arch of atlas, etc)/infective (tuberculosis)/inflammatory (Rheumatoid affection of the C1-C2 complex) conditions. We had five patients who had intra-ligamentous injuries but all had some or other of the above associated conditions and so was excluded from the present study. We agree that pure ligamentous injuries are more prone to persistent instability^11^ rather than avulsion fractures associated with C1 fractures and hence should be operated upon. We treated one case of avulsion fracture conservatively as described above with good results.

C1-C2 rotary subluxation has been extensively reviewed in the literature to be predominantly occurring in children. All our patients were children, unfortunately two of them presented late. Though the recommendation in the literature is predominantly conservative^6^ but most of our patients were Type III and necessitated surgery in the form of C1-C2 posterior fusion [Figure 2].

**Figure 3:**
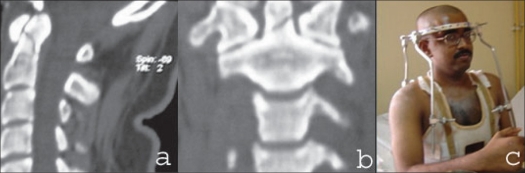
Reconstructed sagittal CT scan (a) and coronal CT scan (b) shows undisplaced type 2 fracture of the odontoid in a 26 years old male. The patients was treated (c) conservatively with halo vest immobilization

**Figure 4:**
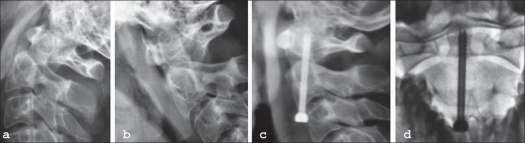
Lateral x-ray upper cervical spine in extension (a) in flexion (b) shows displaced type 2 odontoid fracture. Lateral (c) and AP x-ray (d) of the same patients after anterior odontoid screw fixation

Odontoid fractures have been reported in the literature to be commonest in Type II variety^11^ as also in our series (11 of 13). Undisplaced fractures without significant mobility on flexion/extension X-rays are treated conservatively, usually with a halo vest immobilization [Figure 3]. Displaced, non-comminuted, fresh and reducible Type II fractures were treated with anterior odontoid screw fixation [Figure 4]. We put in a single odontoid screw and found putting in two screws was technically not feasible. The biomechanical^12^ and clinical^13^ studies in literature give ample efficacy of the same. Comminuted, non-reducible and/or late presentation of Type II fractures was treated with posterior instrumented C1-C2 fusion. In two of them we had to resort to wiring technique because the lack of reduction negates an attempt of transarticular screw fixation. In both these, we had to immobilize them postoperatively with SOMI brace for three months. Those who underwent transarticular screw fixation had a very stable construct and did not require postoperative immobilization as suggested by Magerl.^14^ We did not have any serious technique related complications and the contemporary publications also vouch for their safety. Reported incidence of fusion is about 98%^15^ and the incidence of vertebral artery injury is about 2.2% per screw with the incidence of neurological complications being about 0.1%.^16^

The management of Type II injuries still remains controversial in many aspects. Various considerations come into play including the amount of displacement,^17^ age of the patient, presence or absence of comminution, whether the injury is fresh or old and whether there is neurological deficit or not. It must be remembered that neurological deficit is infrequent and occur in males with a smaller spinal canal diameter and who have sustained high velocity injuries.^18^ Normally at this level, one third of the spinal canal (about 3 cm in AP diameter) is occupied by each of the dens, the spinal cord and the free space around^19^ (Steel's rule of three) and hence the rarity of neurological deficits. However progressive myelopathy has been recorded by Crockard^20^ in a small subgroup of patients secondary to displaced, old, untreated or inadequately treated Type II odontoid fractures. They require decompression and occipitocervical fusion. We had two patients in this group, both of them untreated from the time of injury. In one, the compression was from the back only (so posterior decompression and C0-C2 fusion with contoured rod fixation was done) while in the other, compression was from the front and back (so anterior transoral decompression/odontoidectomy with posterior C0-C2 fusion with contoured rod fixation was done in the same sitting). Both improved in their myelopathy by one grade though had some persistent spasticity at the last followup.

**Figure 5:**
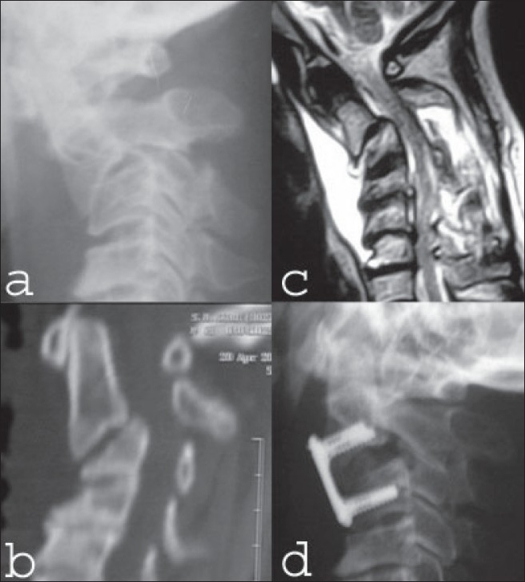
62 years old gentleman presented with C2-C3 bifacetal dislocation three weeks after the injury. Lateral x-rays (a) reconstructed sagittal CT scan (b) shows C2-C3 facetal dislocation. Mid sagittal T2WI of MRI (c) shows C2-C3 subluxation with cord compression. The patieints was treated skull traction to achieve partial reduction. Later on anterior C2-3 discectomy, fusion and fixation with locking plate and screw was done. Post operative lateral x-ray (d) shows reduction, C2-3 fusion and plate application

Hangman fracture, if displaced and angulated (Type IIa and Type III) is usually treated surgically with a recent focus on treating Type IIa surgically,^21^,^22^ which in the past was treated conservatively by many. Surgical options include posterior direct pars/pedicle repair by osteosynthesis with screws and anterior C2/3 discectomy, fusion and fixation with locking plate/screws after reduction by traction, as much as possible. We have no experience with posterior surgery and have treated four patients with anterior C2/3 Anterior Cervical Discectomy & Fusion (ACDF) [Figure 5]. Though it is technically difficult to reach C2 with a short neck and limited extension, it is possible with reasonable safety. Except for one patient of transient swallowing difficulty, we did not have other complications.

## CONCLUSION

A thorough understanding of the common types of upper cervical injuries helps in picking them up early. A detailed clinical and radiological workup including X-rays, CT scans and MRI is mandatory for a complete analysis of the injury. Most of them need good conservative care and few require surgery, which is sometimes technically demanding. All occipital condylar fractures, most of Atlas fractures and quite a few of Axis fractures (except Type II Odontoid and Type IIA/III Hangman varieties) are treated conservatively. Most of Type II Odontoid fractures (if displaced, unstable or old/unreduced varieties producing myelopathy) are treated operatively. Most of Type IIA and all Type III Hangman fractures are treated by surgical methods. The results of conservative and surgical treatment are good and the complication rate is not high. Late presentation is often associated with secondary complications especially persistent pain and myelopathy. Early presentation and diagnosis is the key to the successful outcome of treatment and is vital in preventing secondary complications especially with neural deficit.
